# Partial-Body Irradiation in Patients with Prostate Cancer Treated with IMRT Has Little Effect on the Composition of Serum Proteome

**DOI:** 10.3390/proteomes3030117

**Published:** 2015-06-30

**Authors:** Monika Pietrowska, Karol Jelonek, Joanna Polanska, Anna Wojakowska, Lukasz Marczak, Ewa Chawinska, Aleksanda Chmura, Wojciech Majewski, Leszek Miszczyk, Piotr Widlak

**Affiliations:** 1 Maria Sklodowska-Curie Memorial Cancer Center and Institute of Oncology, Gliwice Branch, Gliwice 44-101, Poland; E-Mails: m_pietrowska@io.gliwice.pl (M.P.); kjelonek@io.gliwice.pl (K.J.); awojakowska@io.gliwice.pl (A.W.); ewa.chawinska@io.gliwice.pl (E.C.); aleksandra.chmura@io.gliwice.pl (A.C.); wmajewski1@poczta.onet.pl (W.M.); leszek@io.gliwice.pl (L.M.); 2 Institute of Automatics Control, Silesian University of Technology, Gliwice 44-100, Poland; E-Mail: joanna.polanska@polsl.pl; 3 Institute of Bioorganic Chemistry, Polish Academy of Sciences, Poznań 61-704, Poland; E-Mail: lukasmar@ibch.poznan.pl

**Keywords:** IMRT, mass spectrometry, prostate cancer, radiation response, serum proteomics

## Abstract

Partial body irradiation during cancer radiotherapy (RT) induces a response of irradiated tissues that could be observed at the level of serum proteome. Here we aimed to characterize the response to RT in group of patients treated because of prostate cancer. Five consecutive blood samples were collected before, during, and after the end of RT in a group of 126 patients who received definitive treatment with a maximum dose of 76 Gy. Serum peptidome, which was profiled in the 2000–16,000 Da range using MALDI-MS. Serum proteins were identified and quantified using the shotgun LC-MS/MS approach. The majority of changes in serum peptidome were detected between pre-treatment samples and samples collected after 3–4 weeks of RT (~25% of registered peptides changed their abundances significantly), yet the intensity of observed changes was not correlated significantly with the degree of acute radiation toxicity or the volume of irradiated tissues. Furthermore, there were a few serum proteins identified, the abundances of which were different in pre-RT and post-RT samples, including immunity and inflammation-related factors. Observed effects were apparently weaker than in comparable groups of head and neck cancer patients in spite of similar radiation doses and volumes of irradiated tissues in both groups. We concluded that changes observed at the level of serum proteome were low for this cohort of prostate cancer patients, although the specific components involved are associated with immunity and inflammation, and reflect the characteristic acute response of the human body to radiation.

## 1. Introduction

Radiotherapy (RT), either alone or in combination with other treatment modalities, is an effective treatment of patients suffering from different types of cancer which allows the delivery of a cytotoxic dose to the tumor while sparing the structure and function of critical healthy organs. Contemporary radiation oncology takes advantage of technologically advanced conformal RT or intensity modulated RT (IMRT), where dose distribution conforms to the tumor shape, allowing a higher dose of radiation to be delivered to the tumor, and intentionally reducing the dose and toxicity of radiation to the surrounding normal tissues [1,2,3]. In recent years, low or locally advanced prostate cancers and head and neck cancers have been among the tumors successfully treated with IMRT [4,5,6,7]. However, the exposure of a large volume of the patient’s body to lower doses of radiation is a potential drawback of conformal RT or IMRT, for which biological and clinical relevance is not clear at the moment [8,9]. Hence, knowledge of molecular factors that determine and reflect the response of the patient’s organism to irradiation during such treatment, especially factors that could be implemented for the monitoring and prediction of the toxic effects of radiotherapy, would be of great importance in clinical practice.

Components of blood proteome and peptidome appear to be a promising source of novel biomarkers of human diseases, and numerous studies using mass spectrometry-based profiling of serum, plasma proteome, and peptidome revealed multi-component signatures with potential applicability in the detection and/or classification of cancer [10,11,12,13,14]. It is noteworthy that several components of proposed cancer signatures, especially those characteristic for advanced cancer, were identified as fragments of blood proteins involved in the general response of patient’s organism to the disease (e.g., inflammatory response). Furthermore, profiling of serum/plasma proteome revealed features that reflected the general reaction of the whole patient’s body to anticancer treatment [15,16,17]. However, only a few published works used serum proteome/peptidome profiling, aiming to detect direct radiation-related effects in locally irradiated cancer patients [18,19]. The first comparative analysis of serum samples collected before, during, and after RT was performed in a group of 68 patients treated due to 18 different malignancies. Patients received total maximum doses in a range of 1.5–86.4 Gy, with the time interval between consecutive blood samples in a range of one to 55 days. In spite of very heterogeneous material, the authors identified clear differences between pre-exposure and post-exposure samples [20]. Another comparative analysis of pre- and post-RT blood samples was performed on a group of larynx cancer patients treated with doses ranging from 51 to 72 Gy, which allowed detection of radiation-related changes in the serum peptidome after the end of RT [21]. Further analysis of IMRT-related effects in a group of patients suffering from head and neck cancer (head and neck squamous cell carcinoma (HNSCC)) revealed that changes in serum peptidome features were associated with intensity of acute mucosal toxicity and were affected by low-to-medium doses delivered to large volumes of normal tissues [22]. More recently, the multi-protein serum signature of response to partial body irradiation during treatment of HNSCC patients was proposed, which included up-regulation of inflammation-related factors, and down-regulation of apolipoproteins and blood coagulation-related factors [23].

In general, changes in blood proteome features depend on doses of radiation and volumes of irradiated tissues (dose-volume effect). In fact, no such changes were detected in patients irradiated locally with low doses (1 Gy dose fractions up to 6 Gy) during non-oncological treatment or in patients irradiated to very small volumes (up to 4 ccm) because of skin cancer, while marked changes were detected several years after low-dose whole-body irradiation in persons involved in radiation accidents [24]. However, it is currently not clear how the specificity of normal tissues irradiated during radiotherapy of differently located tumors (e.g., type and intensity of radiation toxicity) affects the whole body response measured at the level of serum proteome. We aimed here to characterize such a response upon partial body irradiation of patients undergoing IMRT because of prostate cancer. Moreover, changes noted in this group of patients were compared with effects observed in a previously characterized group of patients with HNSCC [22], where radiation doses and volumes of tissues irradiated during IMRT were similar. We concluded that although several radiation-induced effects were detected at the level of serum proteome of prostate cancer patients, their intensities were weaker when compared to the effects observed in the blood of patients irradiated because of HNSCC, which apparently mirrored differences in acute toxicity induced by RT in both groups.

## 2. Experimental Section

### 2.1 Characteristics of Patient Group

One hundred and twenty-six patients with prostate adenocarcinomas were enrolled into this prospective study; all participants were Caucasians aged 49–84 years (median age 69 years). Primary tumor stage was scored as: T1 (43%), T2 (44%), T3 (11%), and T4 (2%); 95% of N0; all M0. All patients were subjected to IMRT using 6 megavolt photons with two Gy daily fraction doses according to the conventional five-times-a-week irradiation scheme. Total radiation doses delivered to gross tumor volume (GTV) were in the 74–76 Gy range (median 76 Gy), overall treatment time was in the 50–85 day range (median 54 days). Sixty-six patients (52%) from the high-risk group were also irradiated at the pelvic lymph nodes at 44 Gy total dose. All patients were subjected to computer tomography (CT) during RT planning at 2–4 weeks (median 16 days) before start of the treatment. Furthermore, treatment was monitored on a daily basis using fiducial-based image-guided RT (IGRT). Androgen deprivation therapy was applied to 85% of patients before and/or during RT; none of the patients enrolled in the study was subjected to prostatectomy. Gastrointestinal toxicity and genitourinary toxicity reactions were assessed every week during the RT according to the RTOG/EORTC (Radiation Therapy Oncology Group / European Organization for Research and Treatment of Cancer) protocol. Five consecutive blood samples (5 mL each) were collected from each patient: pre-treatment sample A (2–5 weeks before RT and also before CT; pre-CT/RT); pre-treatment sample B (usually directly before the start of RT and about two weeks after CT; post-CT/pre-RT); within-treatment sample C (16–47 days after the start of RT; median 25 days); post-treatment sample D (always on the last day of the treatment); post-treatment sample E (21–75 days after the end of RT; median 35 days). The study was approved by the appropriate ethics committee and all participants provided informed consent indicating their conscious and voluntary participation.

### 2.2 Profiling of the Low-Molecular-Weight Fraction of Serum Proteome by MALDI-ToF Mass Spectrometry

Blood samples collected into Vacutainer tubes were incubated for 30 min at room temperature, then centrifuged at 1000 g for 10 min to remove clots; resulting sera were portioned and stored at −70 °C. Before analysis, samples were diluted 1:5 with 20% acetonitrile (ACN) and 25 mM ammonium bicarbonate, and centrifuged though Microcon 30 kDa cut-off filters (EMD Milipore, Billerica, MA, USA) at 3000 g for 30 min to remove large-molecular-weight components; filtered samples were concentrated ~3 times by drying in SpeedVac and stored at −70 °C until measurement. The analysis was performed using the Autoflex MALDI-ToF/ToF mass spectrometer (Bruker Daltonik GmbH, Bremen, Germany). The analyzer worked in the mass range between 2000 and 16,000 Da, in a positive linear mode. Each sample was passed repeatedly 15 times through ZipTip C18 tip-microcolumns to saturation, then washed with a water solution of 0.1% trifluoroacetic acid (TFA), and eluted with 1 μL of saturated sinapinic acid in 50%ACN/0.1%TFA (matrix solution) onto the 600 μm AnchorChip plates (Bruker Daltonik GmbH, Bremen, Germany). The ZipTip purification procedure was repeated twice for each sample and for each spot on the plate two spectra were recorded after 200 laser shots (*i.e.*, four spectra were recorded for each sample); all samples were analyzed in random sequence to avoid batch effect. Mass calibration was performed after every four samples using protein standards in the range of 2.8 to 16.9 kDa (Protein Calibration Standard I; Bruker, (Bruker Daltonik GmbH, Bremen, Germany).

### 2.3 Processing of MALDI Spectra and Statistical Analyses

The pre-processing of spectra, which included baseline removal, alignment, and averaging of technical repeats, as well as the normalization of the total ion current (TIC), was performed according to standard procedures [25]. In the second step, the spectral components, which reflected [M + H]^+^ peptide ions recorded at defined *m/z* values, were identified using decomposition of mass spectra into their Gaussian components followed by several post-processing steps as described in more detail elsewhere [26,27]. The average spectrum was decomposed into a sum of Gaussian bell-shaped curves by using a variant of the expectation maximization (EM) algorithm and Bayesian Information Criterion (BIC) for model selection. The initial set of Gaussian components was further processed to merge overlapping components (homogenous in variance and with main values closer than 0.1% of the *m/z* value) and to remove those presumably representing the residual baseline (components with a coefficient of variation bigger than 25%), which resulted in dimension reduction to 218 Gaussian components. This final set of Gaussians was used to compute features of registered spectra (termed spectral components afterward) for all samples by the operations of convolutions with Gaussian masks. For each spectral component, the normality of distribution of changes was assessed using the Lilliefors test to provide optimal tools for statistical analysis. Either the t-test or the Wilcoxon test was applied for the analysis of changes in abundances of spectral components. The correlation between abundance of spectral components and volume of irradiated tissue was analyzed using either Pearson’s correlation coefficient or Spearman’s rank correlation coefficient (depending on the type of distribution). Differences between sub-groups of patients were analyzed using either t-test or U-test (depending on the type of distribution). *p*-value equal to 0.05 was considered as a standard significance threshold level in all cases. Since the overall changes in peptidome profiles were under consideration, instead of the correction of each *p*-value for multiple testing, the number of significantly changed components was accompanied by the false discovery rate (FDR) estimation.

### 2.4. Identification of Serum Proteins by LC-MS/MS

Serum samples (~15 μg of proteins) were reduced with 5 mM dithiothreitol (5 min at 95 °C), alkylated with 10 mM iodoacetamide (20 min in darkness at room temp.), and then digested with trypsin (overnight at 37 °C; 1:100 enzyme to protein ratio). The analysis was performed on Dionex UltiMate 3000 RSLC nanoLC System connected to a Q Exactive Orbitrap mass spectrometer (Thermo Scientific, Waltham, MA, USA). Tryptic peptides were separated on reverse phase Acclaim PepMap RSLC nanoViper C18 column (75 μm × 25 cm, 2 μm granulation, Thermo Scientific, Waltham, MA, USA) using 240 min acetonitrile gradient (from 4% to 60%, in 0.1% formic acid) at 30 °C and a flow rate of 250 nL∙min^−1^. The spectrometer was operating in the data-dependent MS/MS mode with survey scans acquired at a resolution of 70,000 at *m/z* 200 Da in MS mode and 17,500 at *m/z* 200 Da in MS^2^ mode, respectively. The spectra were recorded in the scan *m/z* range 300–2000 in the positive ion mode. Higher energy collisional dissociation (HCD) ion fragmentation was performed with normalized collision energies set to 25. Protein identification was performed using the UniProt/SwissProt human database [28] with a precision tolerance of 10 ppm for peptide masses and 0.05 Da for fragment ion masses. Protein hit was considered significant only when based on at least two unique peptide hits or unique peptides covering at least 2% of the protein sequence, and the value of posterior error probability (PEP) was lower than 0.05. The abundances of identified proteins were estimated using the MaxQuant software [29]. Only proteins quantified above the threshold in at least 80% of samples in each group were analyzed for significance of changes using the t-test or the Wilcoxon signed rank test (depending on normality of data). The nearest neighbors method, with standardized Euclidean distance metric span over all proteins and k equal to 3, was used for missing data imputation.

## 3. Results

### 3.1 Profile of Endogenous Serum Peptidome Was Affected in Patients Irradiated Due to Prostate Cancer

**Figure 1:**
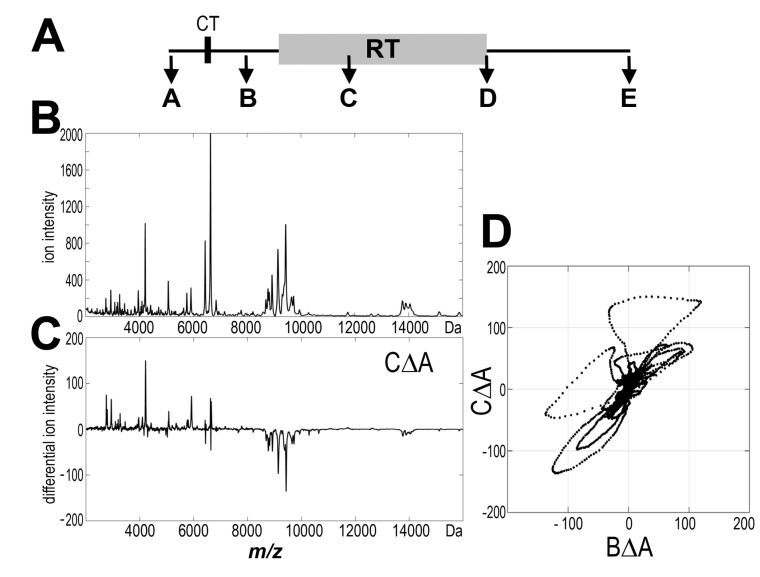
MALDI-based serum peptidome profiling. (**A**) Flow chart of the blood sample collection; (**B**) Average mass spectrum registered in the range of 2000 to 16,000 Da for pre-treatment samples; (**C**) Average differential spectrum for individual pre-treatment A and within-RT C samples; (**D**) Scatterplot of pair-wise comparison of differential spectrum CΔA *versus* differential spectrum BΔA.

The general influence of radiotherapy on mass profiles of the low-molecular-weight fraction of serum proteome (*i.e.*, endogenous peptidome) was analyzed in the first step. Figure 1A shows a time chart of the study. Mass profiles were established for five consecutive serum samples collected before any radiation treatment (sample A), after CT (and before the start of RT; sample B), and during RT (after delivery of about half of a total dose; sample C), as well as immediately after and about one month after the end of RT (samples D and E, respectively). Figure 1B shows an average MALDI-ToF spectrum of serum peptidome registered in the 2000 to 16,000 Da range for prostate cancer patients; 218 components (corresponding to peptide ions) were distinguished in this mass range (detailed information on abundances of detected components and their changes during RT are shown in Supplementary Table S1). For each pairwise analysis, individual differential spectrum was obtained (see Figure 1C), then the statistical significance of differences in abundance of each peptide was estimated in the whole group of patients between compared time points. Table 1 (column RT_1 + 2) shows numbers of components that changed their abundances significantly (*p* < 0.05) between compared time points. The highest number of differences was observed when pre-treatment and within-RT samples were compared (the AΔC change), where ca. 27% of registered peptide ions changed their abundance significantly. Some statistically significant changes were also observed when pre-treatment samples and both post-RT samples were compared (the AΔD and AΔE change, respectively). In contrast, statistically significant changes were not observed when pre-treatment and post-CT samples were compared (the AΔB change) (see Figure 1D). Among 58 components that changed their abundance between pre-treatment and within-RT samples (the AΔC change), 26 components were up-regulated (mostly *m/z* values below 5000 Da) while 32 components were down-regulated (mostly *m/z* values above 7000 Da) in response to radiation. In most cases, the AΔC changes were reversed/compensated in post-RT samples D and E.

**Table 1:** Numbers of peptide serum components, whose levels changed significantly (*p* < 0.05) between analyzed time points in the all-patient group (RT_1 + 2) or in subgroups with different RT schemes (RT_1 and RT_2, also see Figure 3B); corresponding FDR level estimates are showed in square parentheses.

Change/Patient Group	RT_1 + 2 (*n* = 126)	RT_1 (*n* = 60)	RT_2 (*n* = 66)
AΔB	12 (92%)	4 (100%)	10 (100%)
AΔC	58 (19%)	57 (19%)	41 (27%)
AΔD	33 (33%)	19 (58%)	18 (61%)
AΔE	31 (35%)	29 (38%)	21 (52%)

**Figure 2:**
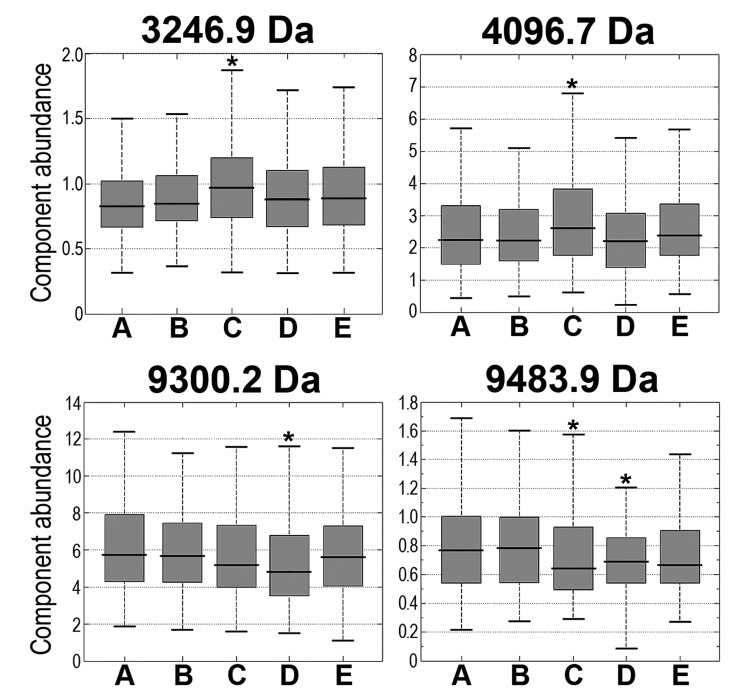
Examples of registered peptide ions, whose abundances were affected in the serum of irradiated patients. Each boxplot shows: minimum, lower quartile, median, upper quartile, and maximum values; consecutive samples are marked A, B, C, D, and E; asterisks marked significant differences comparing to pre-treatment sample A (*p* < 0.05).

Figure 2 shows examples of serum components (peptide ions) that changed their abundance in analyzed samples. It is noteworthy that no statistically significant correlation between the intensity of RT-induced changes in serum peptidome and the PSA level (neither pre-treatment nor post-treatment) was detected. Moreover, similar levels of RT-induced changes were observed in the serum of patients who either received or did not received androgen deprivation therapy. We concluded that partial body irradiation during the treatment of prostate cancer induced changes in abundance of several components of serum peptidome, which were detected primarily in samples collected during radiotherapy. However, there was only a moderate significance of detected changes, which was further reduced in samples collected after the end of radiotherapy.

### 3.2 Changes in Serum Peptidome Were Associated with Neither Volume Nor Toxicity of Irradiated Tissue

**Figure 3:**
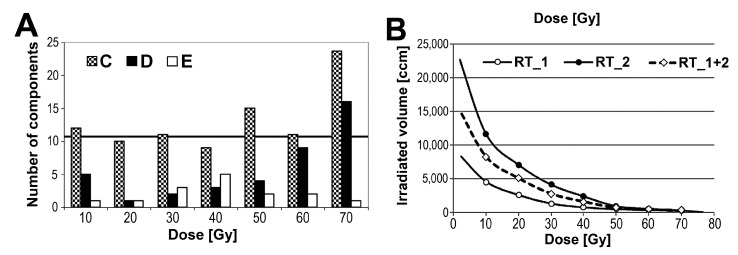
Dose-volume effect in radiation response. (**A**) Numbers of peptidome components in within- and post-RT samples (C, D, and E, respectively) that correlated with volume of tissue irradiated at a given dose (*p* < 0.05) in the all-patient group; horizontal line represents a threshold corresponding to FDR = 100%; (**B**) Averaged dose-volume histograms for the all-patient group (RT_1 + 2) and subgroups of patients with two different treatment plans (RT_1 and RT_2).

The same maximum GTV dose (76 Gy) was planned for practically all patients in the analyzed group. However, because of the variability of individual treatment plans, different dose-volume histograms were established. Hence, in the next step, we searched for correlations between abundances of peptidome components and the volume of tissue irradiated with different doses (every 10 Gy up to 70 Gy). Figure 3A shows the numbers of components, whose abundances in within- and post-RT serum samples were correlated with volumes of tissues irradiated with a given dose (*p* = 0.05 was selected as a significance threshold) (see Supplementary Table S2 for details). We found that only when tissue volume irradiated with a 70 Gy dose (*i.e.*, volume corresponding to the tumor target) was analyzed, the observed number of correlated components was higher than expected by chance: there were 20 correlated components in sample C collected during RT (estimated FDR = 55%) and 16 correlated components in sample D collected immediately after the end of RT (estimated FDR = 69%). However, when larger volumes of normal tissues irradiated with lower doses were considered, the number of observed significant correlations was generally lower than expected by chance. To further verify the possibility that the volume of tissues irradiated with low-to-medium doses did not effect features of the serum peptidome, two subgroups of patients whose normal tissues were irradiated with different doses were compared. In the first subgroup (RT_1), the planned maximum dose was delivered to the cancer target only, while in the second subgroup (RT_2), an additional 44 Gy dose was delivered to the pelvic lymph nodes. As a result, significantly larger volumes of normal tissues were irradiated with doses up to 44 Gy in the latter group of patients (about three-fold higher, on average; see Figure 3B). The numbers of components that changed their abundances in samples collected in both subgroups are shown in Table 1 (columns RT_1 and RT_2, respectively). Similar to the all-patient group, in both compared subgroups the highest number of significant changes was observed when pre-treatment and within-RT samples were compared (the number of effected components appeared slightly higher in the RT_1 subgroup than in RT_2 one). Furthermore, an abundance of each peptidome component was compared directly between both subgroups of patients. Importantly, statistically significant differences were not detected when abundances of serum components were compared for within-RT and post-RT samples. Hence, we concluded that irradiation of normal tissues with clinically low-to-medium doses (<40 Gy) did not reveal a significant impact on features of serum peptidome of patients irradiated locally due to prostate cancer treatment.

In the last step, we searched for potential associations between the features of the serum peptidome and the intensity of acute toxicity induced by the treatment. In general, IMRT was well-tolerated in the analyzed group, and very few patients showed a high degree of gastrointestinal or genitourinary toxicity (for only six patients a toxicity grade of 3 RTOG/EORTC points was recorded). Hence, patients were divided into two subgroups: (I) low-grade toxicity group (toxicity grade higher than 1 point was not recorded during RT; *n* = 92), and (II) medium/high-grade toxicity group (toxicity grade was higher than 1 point at least once during RT; *n* = 34). Importantly, statistically significant differences were not observed between these subgroups when abundances of the serum components were compared in within-RT and post-RT samples (*i.e.*, the observed number of differences was similar to that expected by chance). Hence, we concluded that low and moderate toxicity induced by IMRT in prostate cancer patients did not significantly affect features of the serum peptidome. Nevertheless, one should note that the assessment of the toxicity of RT is rather subjective in prostate cancer patients and does not directly reflect the state of irradiated epithelium (unlike an assessment of the mucosa reaction in head and neck cancer patients).

### 3.3 Radiotherapy of Prostate Cancer-Effected Levels of a Few Serum Proteins

**Figure 4:**
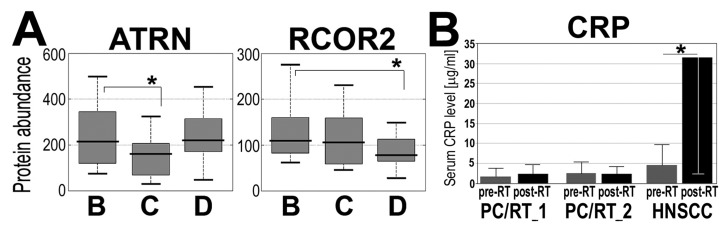
Examples of proteins, whose abundances were affected in serum of irradiated patients. (**A**) Proteins analyzed by LC-MS/MS; each boxplot shows: minimum, lower quartile, median, upper quartile, and maximum values; (**B**) Pre-RT and post-RT levels of CRP assayed in two subgroups of patients with prostate cancer (RT_1 and RT_2) and patients with HNSCC, showed are mean values ± S.D. Asterisks marked statistical significance (*p* < 0.05) when changes between pre-RT and within-RT or post-RT samples were analyzed (samples B, C, and D, respectively, in panel A).

In the second part of the study, we identified and quantified serum proteins using the so-called shotgun approach. Twenty patients were selected from the RT_2 group, and then 20 sets of pre-RT, within-RT, and post-RT sera (samples B, C, and D, respectively) were analyzed using the LC-MS/MS technique. In general, abundances of 137 out of about 400 identified serum proteins were used for estimation of radiotherapy-induced changes (including 39 immunoglobulins and Ig-related proteins) (list of all quantified proteins is presented in the Supplementary Table S3). We found that 10 serum proteins changed their levels significantly (*p* < 0.05) when within-RT or post-RT samples were compared to pre-RT samples (Table 2). Several different modes of radiation-induced changes were observed (Figure 4A). Noticeably, proteins associated with inflammation and immune response (attractin, apolipoprotein A-II, complement C6, lipopolysaccharide-binding protein, and immunoglobulins) were among those affected by RT. Similar analysis performed in pre-RT and post-RT serum samples collected from patients irradiated due to HNSCC revealed many more proteins (about 100 species, including immunoglobulins and Ig-related proteins) affected by radiation, and a large fraction of them was associated functionally with immunity, acute phase, and inflammation [23]. Additionally, we compared serum levels of C-reactive proteins (CRP) assessed by immunonephelometry in pre-RT and post-RT samples collected in groups of patients treated because of prostate cancer and because of HNSCC. As one would expect, a significant RT-induced increase of the CRP level was noted in HNSCC patients but not in prostate cancer patients (Figure 4B). We concluded that although changes in abundances of serum proteins associated with immunity and inflammation were characteristic for the response of the human body to ionizing radiation, the extent of the changes induced by RT in blood of prostate cancer patients is relatively low, which corresponds to the low acute toxicity induced by IMRT in analyzed group of patients.

**Table 2:** Proteins whose levels in serum of prostate cancer patients were effected upon IMRT.

Protein ID	Protein Name	BΔCΔD Change	BΔC *p*-Value	BΔD *p*-Value	CΔD *p*-Value
P18428	Lipopolysaccharide-binding protein	u/d	0.010	0.432	0.003
P68871	Hemoglobin subunit beta (HBB)	u/d	0.661	0.0522	0.019
P01767	Ig heavy chain V-III region BUT	u/d	0.108	0.737	0.042
Q68CK4	Leucine-rich alpha-2-glycoprotein	u/n	0.064	0.035	0.217
P13671	Complement C6 (CO6)	u/n	0.084	0.033	0.785
P01598	Ig kappa chain V-I region EU	n/u	0.263	0.028	0.157
O75882	Attractin (ATRN)	d/u	0.101	0.698	0.049
Q8IZ40	REST corepressor 2 (RCOR2)	d/d	0.194	0.011	0.130
P02652	Apolipoprotein A-II (APOA2)	d/d	0.145	0.049	0.550
P02776	Platelet factor 4 (PLF4)	d/n	0.007	0.015	0.765

Showed are modes of changes between pre-RT sample B, within-RT sample C, and post-RT sample D (d—down-regulation, u—up-regulation, n—neutral), and significance of changes between compared samples.

## 4. Discussion

There are several reports documenting the effects of partial body irradiation during cancer radiotherapy at the level of the blood proteome [20,23,30,31] and the endogenous serum peptidome [20,21,22]. Such molecular changes observed in the blood of cancer patients apparently reflected the pleiotropic effect of radiation on both tumor target and normal tissues irradiated with lower doses due to characteristics of conformal radiotherapy. Molecular changes observed during and upon completion of radiotherapy could be associated with different processes accompanying the treatment, which include tumor regression, toxic effects of radiation, and healing of the acute reactions. In fact, the most abundant groups of serum proteins (and/or their endogenous fragments) whose changed levels were observed in the blood of irradiated humans consisted of factors involved in acute phase, inflammation, innate immunity, and immune response [18,19,20,21,22,23,24,30,31]. These processes are involved not only in the human body response to malignancy, but also play a role in the developing and healing of radiation toxicity [32]. However, even though changes in the blood proteome reflect general processes involved in the whole-body response to the treatment, their particular features could be determined by radiation dose-volume effect and the specific radiosensitivity of irradiated tissue.

We have previously performed an in-depth characterization of the serum endogenous peptidome of patients treated with IMRT because of HNSCC. The analyzed group consisted of 72 patients treated with continuous accelerated RT (1.8 Gy daily fraction dose), who received total doses in a range of 66.6–73.8 Gy (however, similar effects were observed in a group of HNSCC patients treated with a conventional five-times-a-week fractionation scheme; data not published). Massive changes in serum peptidome profiles were observed after the end of treatment: about 45% of registered peptidome components changed their abundance at very high levels of statistical significance (corrected *q* < 0.1%) when pre-treatment samples and samples collected one month after the end of RT were compared. Importantly, the majority of RT-induced changes were compensated/reversed when samples collected after a longer follow-up were analyzed. Furthermore, an apparent correlation between serum peptidome features and the volume of tissues irradiated with low-to-medium doses was established, and several RT-induced changes were associated with the intensity of acute mucosal toxicity [22]. Interestingly, when features of the serum lipidome were analyzed in the same group of HNSCC patients, an obvious impact of the large volume of tissue irradiated with low/medium doses and an association of lipidome changes with acute mucosal reactions were also observed [33]. Here we characterized proteomic profiles in the blood of patients treated because of prostate cancer. IMRT-induced changes observed at the level of the serum peptidome were apparently less intense than in a group of HNSCC patients: about 26% of registered peptidome components changed their abundance at moderate levels of statistical significance (*p* < 0.05, estimated FDR = 19%) when pre-treatment samples and samples collected after three to four weeks of RT were compared. Moreover, neither the volume effect of tissues irradiated with low/medium doses nor the effect related to acute radiation toxicity was detected in this group when features of serum peptidome were analyzed. It is also noteworthy that the exposure of prostate cancer patients to low doses (about 0.2 Gy) during computed tomography performed for RT planning did not reveal any measurable changes when pre-treatment and post-CT/pre-RT samples were compared. Significant differences between IMRT-treated prostate cancer patients and HNSCC patients were detected also when identified serum proteins were quantified in pre-RT and post-RT samples [23]. In both groups of patients, similar dose-volume histograms were assumed during the planning of IMRT. In the group of HNSCC patients, average volumes of tissues irradiated with 10, 20, 40, and 60 Gy doses were 4200, 2800, 1300, and 300 ccm, respectively, which corresponded to the average dose-volume histogram in the subgroup of prostate cancer patients without pelvic lymph node irradiation (subgroup RT_1; see Figure 3B). Hence, apparent differences observed between these groups of patients could be associated with specific features of irradiated tissues rather than with a general dose-volume effect.

IMRT is currently among the main treatment options for prostate cancer patients and HNSCC patients because of its potential to preserve the structure and function of a target organ. However, aggressive treatment (e.g., dose-escalated hypofractionation, accelerated fractionation, or chemoradiotherapy) is usually required in advanced cancer cases, which might be associated with increased risk of treatment toxicity [34,35]. Mucositis is the major symptom of acute radiation toxicity in HNSCC patients, and severe acute mucosal reaction (AMR) can significantly affect the quality of life of patients (causing treatment discontinuation in extreme cases) [36,37,38]. Most importantly, acute mucosal toxicity is usually associated with inflammation and acute phase response [39,40], which could be apparently reflected at the level of the serum proteome [22,23]. In contrast to rather frequent acute toxicity in HNSCC patients, properly planned, image-guided IMRT is well-tolerated in the majority of prostate cancer patients [6,7,41]. Improved outcome of contemporary radiotherapy in prostate cancer patients resulted from the enhanced protection of major organs at risk: bladder and rectum (dose constraints usually considered for these structures strictly depend on the fractionation schedule and do not allow for substantial radiation-induced damage of uroepithelium and intestinal epithelium). Hence, differences in toxicity of IMRT in HNSCC patients and prostate cancer patients could reflect the better protection of healthy organs/tissues in the at-risk area that was possible in the latter group. Nevertheless, statistically significant up-regulation of Interferon-gamma and Interleukin-6 inflammatory cytokines was previously revealed in the blood of patients undergoing IMRT because of prostate cancer, while levels of Interleukin-1 and Interleukin-2 correlated with the risk of radiation toxicity in the analyzed group of 42 patients [30]. Hence, involvement of inflammation-related factors appears to be a general feature of response to irradiation.

We postulated that radiation-induced changes detected at the level of the serum proteome were predominated by factors associated with the toxicity of the treatment. We concluded that the relatively low extent of RT-mediated effects observed in the analyzed cohort of prostate cancer patients presumably reflected good tolerance of the treatment and low radiation toxicity induced by IMRT in this group.

## 5. Conclusions

One should assume that radiotherapy-induced changes observed at the level of serum proteome reflected general response of patient’s body to radiation damage, including inflammation and acute phase response. Hence, we concluded that low extent of radiation-induced changes found at the level of serum proteome in analyzed cohort of prostate cancer patients was probably associated with treatment tolerance and low acute radiation toxicity observed in this group.

## Supplementary Materials

Supplementary File 1

## References

[1] Deasy J.O., Fowler J.F., Mundt A.J., Roeske J.C. (2005). Radiobiology of imrt. Intensity Modulated Radiation Therapy: A Clinical Perspective.

[2] Halperin E.C., Perez C.A., Brady L.W. (2008). Perez and Brady's Principles and Practice of Radiation Oncology.

[3] Brahme A., Lind B.K. (2010). A systems biology approach to radiation therapy optimization. Radiat. Environ. Biophys..

[4] Bourhis J., Overgaard J., Audry H., Ang K.K., Saunders M., Bernier J., Horiot J.C., le Maitre A., Pajak T.F., Poulsen M.G. (2006). Hyperfractionated or accelerated radiotherapy in head and neck cancer: A meta-analysis. Lancet.

[5] Skladowski K., Maciejewski B., Golen M., Tarnawski R., Slosarek K., Suwinski R., Sygula M., Wygoda A. (2006). Continuous accelerated 7-days-a-week radiotherapy for head-and-neck cancer: Long-term results of phase iii clinical trial. Int. J. Radiat. Oncol. Biol. Phys..

[6] Cahlon O., Zelefsky M.J., Shippy A., Chan H., Fuks Z., Yamada Y., Hunt M., Greenstein S., Amols H. (2008). Ultra-high dose (86.4 Gy) IMRT for localized prostate cancer: Toxicity and biochemical outcomes. Int. J. Radiat. Oncol. Biol. Phys..

[7] Spratt D.E., Pei X., Yamada J., Kollmeier M.A., Cox B., Zelefsky M.J. (2013). Long-term survival and toxicity in patients treated with high-dose intensity modulated radiation therapy for localized prostate cancer. Int. J. Radiat. Oncol. Biol. Phys..

[8] De Neve W., de Gersem W., Madani I. (2012). Rational use of intensity-modulated radiation therapy: The importance of clinical outcome. Semin. Radiat. Oncol..

[9] Zwicker F., Swartman B., Roeder F., Sterzing F., Hauswald H., Thieke C., Weber K.J., Huber P.E., Schubert K., Debus J. (2014). *In vivo* measurement of dose distribution in patients’ lymphocytes: Helical tomotherapy *versus* step-and-shoot imrt in prostate cancer. J. Radiat. Res..

[10] Hanash S. (2003). Disease proteomics. Nature.

[11] Aebersold R., Mann M. (2003). Mass spectrometry-based proteomics. Nature.

[12] Wulfkuhle J.D., Liotta L.A., Petricoin E.F. (2003). Proteomic applications for the early detection of cancer. Nat. Rev. Cancer.

[13] Liotta L.A., Ferrari M., Petricoin E. (2003). Clinical proteomics: Written in blood. Nature.

[14] Cho W.C., Cheng C.H. (2007). Oncoproteomics: Current trends and future perspectives. Expert Rev. Proteomics.

[15] Solassol J., Jacot W., Lhermitte L., Boulle N., Maudelonde T., Mange A. (2006). Clinical proteomics and mass spectrometry profiling for cancer detection. Expert Rev. Proteomics.

[16] Palmblad M., Tiss A., Cramer R. (2009). Mass spectrometry in clinical proteomics—From the present to the future. Proteomics Clin. Appl..

[17] Pietrowska M., Widlak P. (2012). Maldi-ms-based profiling of serum proteome: Detection of changes related to progression of cancer and response to anticancer treatment. Int. J. Proteomics.

[18] Marchetti F., Coleman M.A., Jones I.M., Wyrobek A.J. (2006). Candidate protein biodosimeters of human exposure to ionizing radiation. Int. J. Radiat. Biol..

[19] Guipaud O. (2013). Serum and plasma proteomics and its possible use as detector and predictor of radiation diseases. Adv. Exp. Med. Biol..

[20] Menard C., Johann D., Lowenthal M., Muanza T., Sproull M., Ross S., Gulley J., Petricoin E., Coleman C.N., Whiteley G. (2006). Discovering clinical biomarkers of ionizing radiation exposure with serum proteomic analysis. Cancer Res..

[21] Widlak P., Pietrowska M., Wojtkiewicz K., Rutkowski T., Wygoda A., Marczak L., Marczyk M., Polanska J., Walaszczyk A., Dominczyk I. (2011). Radiation-related changes in serum proteome profiles detected by mass spectrometry in blood of patients treated with radiotherapy due to larynx cancer. J. Radiat. Res..

[22] Widlak P., Pietrowska M., Polanska J., Rutkowski T., Jelonek K., Kalinowska-Herok M., Gdowicz-Klosok A., Wygoda A., Tarnawski R., Skladowski K. (2013). Radiotherapy-related changes in serum proteome patterns of head and neck cancer patients; the effect of low and medium doses of radiation delivered to large volumes of normal tissue. J. Transl. Med..

[23] Widlak P., Jelonek K., Wojakowska A., Pietrowska M., Polanska J., Marczak Ł., Miszczyk L., Składowski K. (2015). Serum proteome signature of radiation response: Upregulation of inflammation-related factors, and downregulation of apolipoproteins and coagulation factors in cancer patients subjected to radiotherapy—A pilot study. Int. J. Radiat. Oncol. Biol. Phys..

[24] Nylund R., Lemola E., Hartwig S., Lehr S., Acheva A., Jahns J., Hildebrandt G., Lindholm C. (2014). Profiling of low molecular weight proteins in plasma from locally irradiated individuals. J. Radiat. Res..

[25] Hilario M., Kalousis A., Pellegrini C., Muller M. (2006). Processing and classification of protein mass spectra. Mass Spectrom. Rev..

[26] Pietrowska M., Marczak L., Polanska J., Behrendt K., Nowicka E., Walaszczyk A., Chmura A., Deja R., Stobiecki M., Polanski A. (2009). Mass spectrometry-based serum proteome pattern analysis in molecular diagnostics of early stage breast cancer. J. Transl. Med..

[27] Pietrowska M., Polanska J., Suwinski R., Widel M., Rutkowski T., Marczyk M., Dominczyk I., Ponge L., Marczak L., Polanski A. (2012). Comparison of peptide cancer signatures identified by mass spectrometry in serum of patients with head and neck, lung and colorectal cancers: Association with tumor progression. Int. J. Oncol..

[28] The UniProtKB/Swiss-Prot. http://web.expasy.org/docs/swiss-prot_guideline.html.

[29] MaxQuant, version 1.4.1.1. http://141.61.102.17/maxquant_doku.

[30] Christensen E., Pintilie M., Evans K.R., Lenarduzzi M., Ménard C., Catton C.N., Diamandis E.P., Bristow R.G. (2009). Longitudinal cytokine expression during IMRT for prostate cancer and acute treatment toxicity. Clin. Cancer Res..

[31] Cai X.W., Shedden K., Ao X., Davis M., Fu X.L., Lawrence T.S., Lubman D.M., Kong F.M. (2010). Plasma proteomic analysis may identify new markers for radiation-induced lung toxicity in patients with non-small-cell lung cancer. Int. J. Radiat. Oncol. Biol. Phys..

[32] Atkinson M.J. (2013). Radiation treatment effects on the proteome of the tumour microenvironment. Adv. Exp. Med. Biol..

[33] Jelonek K., Pietrowska M., Ros M., Zagdanski A., Suchwalko A., Polanska J., Marczyk M., Rutkowski T., Skladowski K., Clench M.R. (2014). Radiation-induced changes in serum lipidome of head and neck cancer patients. Int. J. Mol. Sci..

[34] Bourhis J., Etessami A., Wilbault P., Lusinchi A., Calais G., Lapeyre M., Pignon J.P. (2004). Altered fractionated radiotherapy in the management of head and neck carcinomas: Advantages and limitations. Curr. Opin. Oncol..

[35] Behrendt K., Nowicka E., Gawkowska-Suwinska M., Plewicki G., Smolska-Ciszewska B., Giglok M., Suwinski R., Zajusz A. (2014). Early closure of phase ii prospective study on acute and late tolerance of hypofractionated radiotherapy in low-risk prostate cancer patients. Rep. Pract. Oncol. Radiother.

[36] Epstein J.B., Schubert M.M. (2003). Oropharyngeal mucositis in cancer therapy. Review of pathogenesis, diagnosis, and management. Oncology.

[37] Vera-Llonch M., Oster G., Hagiwara M., Sonis S. (2006). Oral mucositis in patients undergoing radiation treatment for head and neck carcinoma. Cancer.

[38] Wygoda A., Maciejewski B., Skladowski K., Hutnik M., Pilecki B., Golen M., Rutkowski T. (2009). Pattern analysis of acute mucosal reactions in patients with head and neck cancer treated with conventional and accelerated irradiation. Int. J. Radiat. Oncol. Biol. Phys..

[39] Sonis S.T. (2004). The pathobiology of mucositis. Nat. Rev. Cancer.

[40] Treister N., Sonis S. (2007). Mucositis: Biology and management. Curr. Opin. Otolaryngol. Head Neck Surg..

[41] Budaus L., Bolla M., Bossi A., Cozzarini C., Crook J., Widmark A., Wiegel T. (2012). Functional outcomes and complications following radiation therapy for prostate cancer: A critical analysis of the literature. Eur. Urol..

